# Convolutional neural network approach for the automated identification of *in cellulo* crystals

**DOI:** 10.1107/S1600576724000682

**Published:** 2024-02-23

**Authors:** Amirhossein Kardoost, Robert Schönherr, Carsten Deiter, Lars Redecke, Kristina Lorenzen, Joachim Schulz, Iñaki de Diego

**Affiliations:** a Sample Environment and Characterization Group, European XFEL GmbH, Holzkoppel 4, 22869 Schenefeld, Schleswig-Holstein, Germany; b Institute of Biochemistry, University of Lübeck, Ratzeburger Allee 160, 23562 Lübeck, Schleswig-Holstein, Germany; c Deutsches Elektronen-Synchrotron DESY, Photon Science, Notkestrasse 85, 22607 Hamburg, Germany; Uppsala University, Sweden; The European Extreme Light Infrastucture, Czechia

**Keywords:** Mask R-CNN, *in cellulo* crystallization, crystal detection, instance segmentation

## Abstract

*In cellulo* crystallization is a rare event in nature. Recent advances that have made use of heterologous overexpression can promote the intracellular formation of protein crystals, but new tools are required to detect and characterize these targets in the complex cell environment. The present work makes use of Mask R-CNN, a convolutional neural network (CNN)-based instance segmentation method, for the identification of either single or multi-shaped crystals growing in living insect cells, using conventional bright field images. The algorithm can be rapidly adapted to recognize different targets, with the aim of extracting relevant information to support a semi-automated screening pipeline, in order to aid the development of the intracellular protein crystallization approach.

## Abstract 2

Open-source deep-learning algorithms (Mask R-CNN) are used to automatically identify intracellular crystals in living insect cell cultures.

## 1. Introduction

Crystallization of proteins in living cells is an emerging field complementing conventional methods of protein crystallization. However, some bottlenecks still limit its broad application. To date, around 80 targets have been found to crystallize in different cells (Tsukimoto *et al.*, 2022 ▸; Li & Cui, 2020 ▸; Nass *et al.*, 2020 ▸; Mudogo *et al.*, 2020 ▸; Schönherr *et al.*, 2018 ▸; Duszenko *et al.*, 2015 ▸; Koopmann *et al.*, 2012 ▸). A number of them were successfully used as targets for protein structure elucidation (Redecke *et al.*, 2013 ▸; Gati *et al.*, 2014 ▸; Sawaya *et al.*, 2014 ▸; Baskaran *et al.*, 2015 ▸; Tsutsui *et al.*, 2015 ▸; Nass *et al.*, 2020 ▸; Lahey-Rudolph *et al.*, 2021 ▸). Intracellular protein crystallization can occur naturally, providing distinct advantages for the cell (Schönherr *et al.*, 2018 ▸), or as a consequence of heterologous gene expression in host cells. Crystallization efficiencies, *i.e.* the percentage of cells containing at least one crystal within the entire cell culture, between more than 80% and less than 1% are observed, and the intracellular crystal size may vary between the sub-micrometre range and several hundred micrometres. Although a correlation with the target protein and the cell type used for gene expression has been shown (Schönherr *et al.*, 2015 ▸, 2018 ▸; Lahey-Rudolph *et al.*, 2020 ▸), the molecular basis remains to be investigated in detail. Targeting the crystals to different cell compartments or modifying the cells or the growth conditions may lead to optimized crystallization efficiency and/or larger crystals (Lahey-Rudolph *et al.*, 2020 ▸; Mudogo *et al.*, 2020 ▸), but identification of small crystals within a few cells of a large population may be required. Even if the resolution of light microscopy allows crystal identification, this can be a time-consuming and laborious task. Thus, methods are required for the automatic characterization, screening and identification of intracellular crystals. Deep-learning-based approaches can aid in this search.

Image segmentation is a method for partitioning an image into multiple disjoint regions via pixel-level classification. This is beneficial to locate objects and object boundaries. The technique is used in object detection and tracking (Maninis *et al.*, 2018 ▸), with downstream applications like action recognition (Khan *et al.*, 2021 ▸), autonomous driving (Kosecka *et al.*, 1998 ▸) or scene understanding (Aarthi & Chitrakala, 2017 ▸). Image segmentation is increasingly used in medical image analysis as well, such as the macro- and microscopic study of blood vessels (Ronneberger *et al.*, 2015 ▸), tumor boundary detection (Havaei *et al.*, 2017 ▸) or neuronal structures (Beier *et al.*, 2017 ▸). Different approaches have been proposed for image segmentation (Sharma *et al.*, 2022 ▸), based on deep-neural networks (He *et al.*, 2017 ▸; Chen *et al.*, 2018 ▸), which produce high-quality results relying on enormous amounts of training data. By utilizing the transfer learning approaches (Pan & Yang, 2010 ▸), it is possible to adapt a heavily, specifically trained model (for solving one problem) to solve a different (but related) problem. This is addressed by training the heavily trained model on a small amount of data on this related problem.

**Figure 1:**
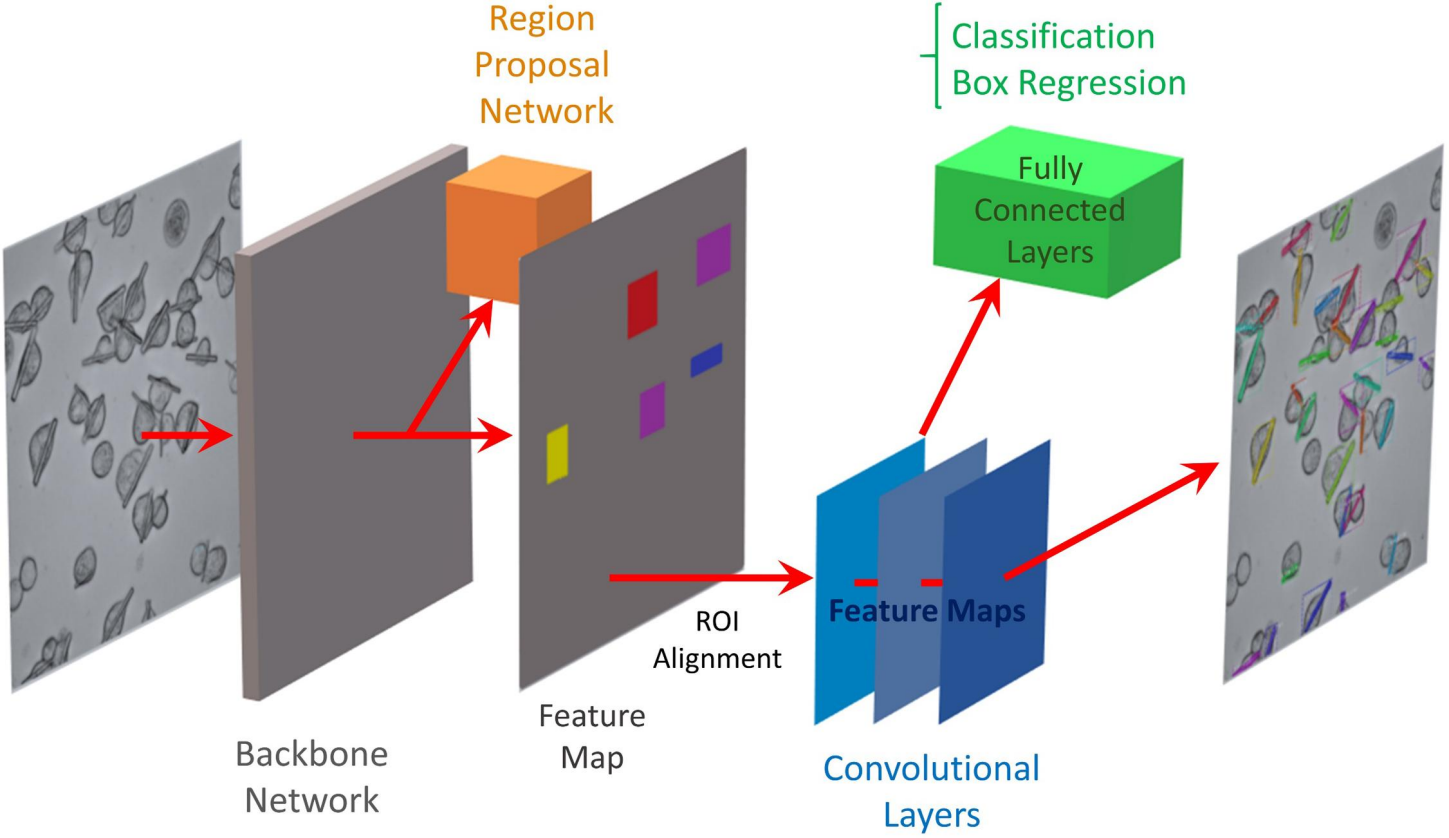
Schematic of the pipeline for training the pre-trained Mask R-CNN model (He *et al.*, 2017 ▸) on our target G. Mask R-CNN adds a branch for object mask prediction, in parallel to bounding box recognition. The model generates bounding boxes, provides the objectness score for detection and accurately segments the intracellular crystals, requiring only a few annotated images as the training dataset.

In this work, we utilized the Mask R-CNN model (He *et al.*, 2017 ▸), an extension of Faster R-CNN (Ren *et al.*, 2017 ▸), adding object mask prediction. Faster R-CNN is a deep convolutional neural network (CNN) for object detection which provides an objectness score for each predicted object. The Mask R-CNN model generates (1) the bounding box around the object instances, (2) the objectness score and (3) the segmentation of the object instance inside the bounding box. The bounding boxes are produced via the region proposal network. The positions are given to the mask generation network to produce pixel-wise segmentation of the objects (Fig. 1 ▸).

Using bright field microscopy images from a crystal containing insect cells, we examined the performance of the Mask R-CNN algorithm, trained on different crystals, to identify different crystal shapes in the cellular environment, as a tool to extract information and aid in the development of protocols for the *in cellulo* crystallography field.

Two different types of *in cellulo* crystals have been used to establish the CNN-based instance segmentation method for protein crystal detection in living cells. The first target, HEX-1 from the fungus *Neurospora crassa*, referred to here as ‘target H’, is a naturally self-assembling protein and main constituent of Woronin bodies in ascomycetes (Tenney *et al.*, 2000 ▸). We recently reported that spontaneous self-assembly of HEX-1 into intracellular crystals is not restricted to the native environment of fungal cells (Lahey-Rudolph *et al.*, 2020 ▸). On infection with a recombinant baculovirus encoding the HEX-1 gene, living insect cells also form micrometre-sized crystals with a hexagonal cross section that enabled structure elucidation using the fixed-target serial femtosecond crystallography approach (FT-SFX) at an X-ray free-electron laser (Lahey-Rudolph *et al.*, 2021 ▸).

The second target, guanosine 5′-monophosphate reductase (GMPR) from the parasite *Trypanosoma brucei*, referred to here as ‘target G’, catalyzes the NADPH-dependent reductive deamination of guanosine 5′-monophosphate to inosine 5′-mono­phosphate, a crucial step in the nucleotide metabolism of the parasite (Hedstrom, 2012 ▸). GMPR has been structurally characterized by conventional X-ray crystallography (Imamura *et al.*, 2020 ▸), but intracellular crystallization has not been reported so far. However, this enzyme shows high structural homology to inosine 5′-monophosphate de­hydrogenase (IMPDH) from *T. brucei*, the structure of which was recently elucidated by SFX using *in cellulo* crystals (Nass *et al.*, 2020 ▸). Since both enzymes are part of the *de novo* purine biosynthesis cycle required for nucleotide production in parasitic protozoa, which differs significantly from that of mammals, these enzymes are considered suitable antiparasitic drug targets (Bessho *et al.*, 2016 ▸).

## 2. Material and methods

### 2.1. Cloning and recombinant baculovirus production

The gene coding for GMPR from *T. brucei* (GenBank Acc. No. XM839789.1) and the Woronin body major protein (HEX-1, GenBank Acc. No. XM_958614) from *N. crassa* were amplified by PCR using primers 5′-CTAGGGTACCTCCTTCAATGAATCGGCATCC-3′ (sense) and 5′-CTAGGCTAGCAAGTTTGGCAACACCGTGAC-3′ (antisense), and 5′-CTAGGGTACCGGCTACTACGACGACGACG-3′ (sense) and 5′-CTAGGC TAGCGAGGCGGGAACCGTGG-3′ (antisense), respectively. The amplicons were cloned into pFastBac1 vector (Thermo Scientific) using KpnI and NheI restriction sites. The standard procedure for the recombinant baculovirus (rBV) generation has been described previously (Lahey-Rudolph *et al.*, 2020 ▸). In brief, recombinant bacmid DNA was generated by the transformation of *Escherichia coli* DH10EmBacY cells (Geneva Biotech), containing a YFP expressing bacmid, with the above-mentioned donor vectors containing the respective (H or G) target. After recombination, the resulting bicistronic bacmid, encoding both YFP and target proteins, was then purified with the ZR Bac DNA Miniprep kit (Zymo Research) and used for lipofection of *Spodoptera frugiperda* Sf9 insect cells with Escort IV reagent (Sigma–Aldrich) according to the manufacturer’s instructions. Virus generation and assembly is then produced inside Sf9 cells. The virus titer of the third-passage (P3) stock was calculated using the TCID50 (tissue-culture infectious dose; Reed & Muench, 1938 ▸) in a serial dilution assay as described previously (Lahey-Rudolph *et al.*, 2020 ▸).

### 2.2. Cell culture, protein production and intracellular crystallization

Insect *Trichoplusia ni* High Five cells (Thermo Scientific) were grown at 27°C and 100 r min^−1^ agitation in ESF921 insect cell-culture medium (Expression Systems), keeping the cell density between 0.4 and 2.5 × 10^6^ cells ml^−1^. For intracellular crystallization of the target proteins, exponentially growing (1 × 10^6^ cells ml^−1^) High Five cells were plated in 2 ml of medium per six-well cell-culture plate and subsequently infected with the respective recombinant P3 baculovirus stock with a multiplicity of infection (MOI) of 0.1. In the late phase of viral infection, High Five cells co-expressed cytosolic YFP in addition to the respective target proteins which were incorporated into crystals. Concretely, after incubation at 27°C for 44–72 h (GMPR) or 72–96 h (HEX-1), the cell cultures were imaged and *in cellulo* crystal formation was verified by light microscopy. For experiments requiring both target crystals to coexist in the same image, cell cultures were co-infected with both viral stocks preserving an MOI of the mixture of 0.1, and images were taken after 72 h post-infection.

### 2.3. Image acquisition parameters

Bright field images (2136 × 2136 px) were captured on a Nikon Qi-2 camera coupled onto a Nikon Ti2-E microscope mounting a Nikon S Plan Fluor ELWD 40× Ph2 ADM (NA = 0.6) objective. The whole process was highly automated, through random multipoint acquisition coupled to autofocusing (employing the Nikon Perfect Focus System) using the NIS *Elements AR* (version 5.41.01) software. Images (corresponding to a 300 × 300 µm field of view) were exported as 8 bit-RGB TIFF format.

**Figure 2:**
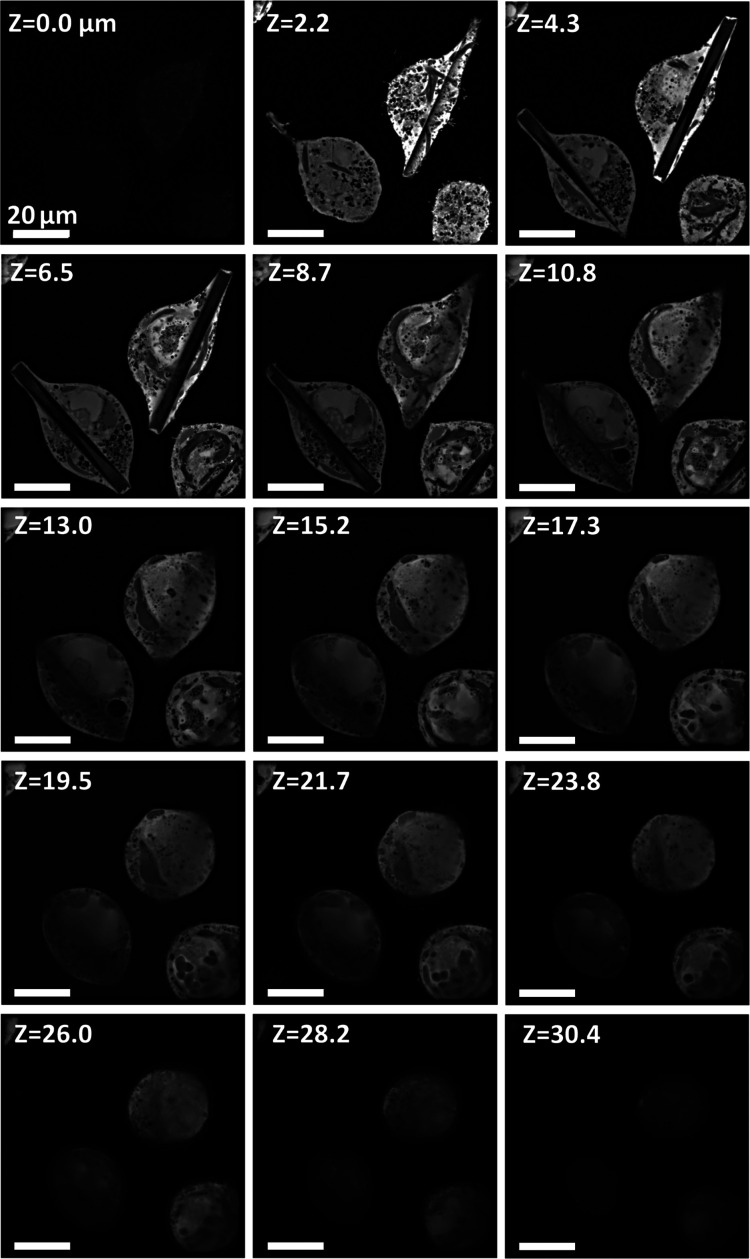
Fluorescence confocal optical sectioning of G-target-containing cells. Fluorescence signal is produced by the co-expressed cytosolic YFP, whereas target G crystals are identified by the absence of signal. A *Z* value of 0 corresponds to the bottom of the cell. The optical sectioning (using 2.1 µm steps) confirms the horizontal position of the crystals at the cell base. All values correspond to micrometre units.

**Figure 3:**
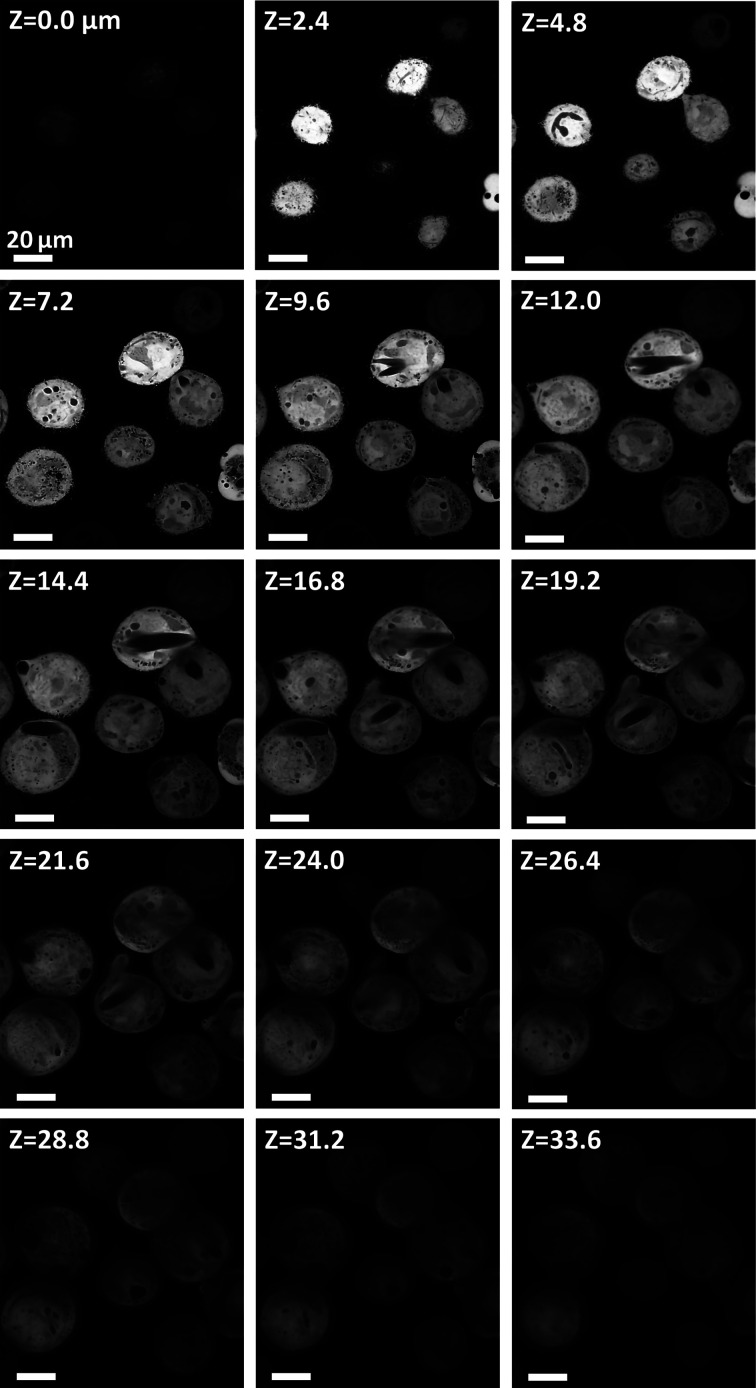
Fluorescence confocal optical sectioning of H-target-containing cells. Fluorescence signal is produced by the co-expressed cytosolic YFP, whereas target H crystals are identified by the absence of signal. A *Z* value of 0 corresponds to the bottom of the cell. The optical sectioning (2.4 µm steps) confirms the relatively random orientation and position of the crystals throughout the cell body, as well as the presence of clustered, multi-shaped crystals. All values correspond to micrometre units.

Optical sectioning of the targets was performed using laser scanning confocal microscopy. Briefly, Z sections of G-target- or H-target-containing cells (Figs. 2 and 3) were taken using a Nikon AX-R confocal microscope, mounting a PlanApo λ 100X oil immersion objective (NA = 1.45), using a pinhole size of 15.9 µm, a 488 laser for excitation and a YFP emission gate (518–551 nm) to detect the co-expressed cytosolic YFP. Through this approach, crystals, which do not contain YFP, appear dark against the fluorescent cell interior. Similar optical Z sections of 2.1 and 2.4 µm thickness were obtained for target G and H, respectively. Images were converted to grayscale TIFF.

### 2.4. Algorithm training and testing

#### 2.4.1. Implementation

The training and test stages were computed with the *SLURM* (Yoo, Jette & Grondona, 2003 ▸) resource management tool on the Maxwell cluster (available at the Deutsches Elektronen-Synchrotron DESY, Hamburg, Germany), incorporating 10 nodes. We used the Matterport Mask R-CNN implementation of Mask R-CNN (https://github.com/matterport/Mask_RCNN). The model was trained on a backbone of ResNet50. We resized and converted the main images to PNG format and 512 × 512 pixel resolution, using the Python Imaging Library (PIL), to reduce the computational complexity of the overall process. Crystals were manually annotated using freeware *Labelme* (Russell *et al.*, 2008 ▸) to produce the ground-truth segmentations to train the Mask R-CNN model.

**Figure 4:**
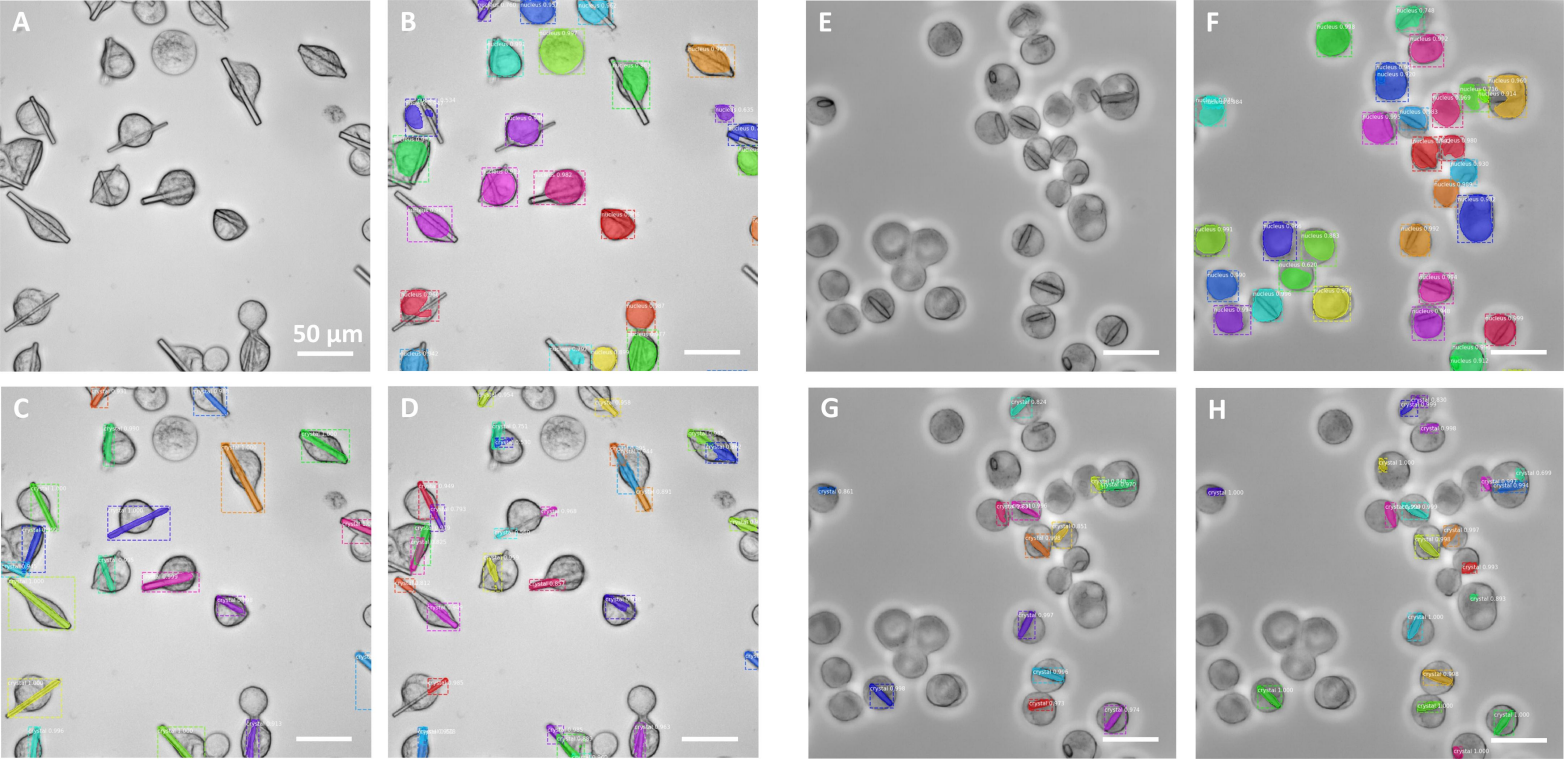
Primary training of the algorithm on 50 images of target G (G_50_, panels C and G) or target H (H_50_, panels D and H), and the evaluation of each of the targets G (left, A–D) or H (right, E–H). Additional training of the algorithm was performed using the data-science-bowl dataset (Caicedo *et al.*, 2019 ▸) for the segmentation of the cell bodies (panels B and F). The results indicate that specific training on the target crystal is required for optimum performance. Performance indicator values are shown in Table 1 ▸.

The annotated images were used for training our models, already pre-trained on the COCO dataset (Lin *et al.*, 2015 ▸). Each of the annotated images consisted of a 300 × 300 µm field of view, with all crystals/cells therein being annotated. In parallel, we trained the Mask R-CNN model with the data-science-bowl dataset (Caicedo *et al.*, 2019 ▸) to detect cell nuclei (Fig. 4). We used the warm-starting strategy to train the Mask R-CNN model on our limited data. The processing details and scripts are available on the shared GitHub page (https://github.com/Amirhk-dev/Convolutional-Neural-Network-Approach-for-the-Automated-Identification-of-in-Cellulo-Crystals). Original and processed images, including annotations, trained models and prediction results, are publicly available for download at Zenodo (https://doi.org/10.5281/zenodo.10475961).

#### 2.4.2. Models trained on single targets

To segment the crystals of target G and H, separate models are trained with 50 images each (resulting in *G*_50_ or *H*_50_ trained models, see Table 1 and Fig. 4). We focused on the respective crystal target for 40 epochs with a batch size of one, with a learning rate (LR) of 1 × 10^−3^ and via the binary cross entropy (BCE) loss function. We chose those parameters by looking at the stabilization of the loss function.

We alternatively trained the pre-trained Mask R-CNN model on 10 and 30 images of each of the targets to study the effect of training on a very small amount of training data (*G*_10_, *G*_30_, *H*_10_ and *H*_30_ trained models, see Table 1), using the same training strategy already described.

To increase the robustness of the model, image augmentation was included, such as random horizontal and vertical flip and cropping of the images.

#### 2.4.3. Incremental learning on targets G and H

To show the generalizability of the trained models on different targets we utilized the incremental learning approach (Geng & Smith-Miles, 2009 ▸). The model trained on 50 images of one target (referred to here as ‘primary’ training, see Table 1) was trained further on a target of a different type (referred to here as secondary training) using 10, 30 or 50 additional images. In this ‘secondary’ training approach, the trained models were tuned with 10 epochs and a learning rate of 1 × 10^−3^, as a standard strategy. To study the effect of learning rate on this secondary training, the smaller value of 1 × 10^−4^ was also investigated.

#### 2.4.4. Single training on combined images of targets G and H

As an alternative to the incremental learning approach, we followed a combined learning strategy (*G*_50_ + *H*_50_), where 100 images (50 from each of the targets) were combined into a single (primary) training set. These images were the same as previously used for the two-stage incremental learning approach (*G*_50_ > *H*_50_). We used the same standard training approach parameters (40 epochs and an LR of 1 × 10^−3^) used for the single-target training.

#### 2.4.5. Evaluation

For each scenario, the performance of the trained models was evaluated on test sets of the two targets, G and H, each containing 150 fully annotated images. We calculated the performance on the basis of three indicators: *F*-measure, Jaccard index and Δ object. The *F*-measure (*F*) is a harmonic mean of the precision (*P*) and recall (*R*), as computed below:

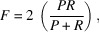

where *P* and *R* are calculated as

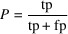

and

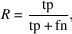

tp, fp and fn being the numbers of true positive, false positive or false negative pixels when comparing the segmentation result with respect to ground-truth segmentation, respectively. The Jaccard index (*J*), on the other hand, is a similarity measure defined as the size of the intersection divided by the size of the union of the sample sets, and it is computed as follows:

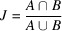

where *A* is the segmentation result and *B* is the ground-truth segmentation.

On the other hand, the Δ object (ΔO) indicates the average absolute difference between the number of objects (crystals) available on the ground-truth image and the number of segmented objects predicted by the trained model (Bideau *et al.*, 2018 ▸):

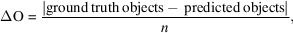


*n* being the total number of images of the dataset.

Note that the *F*-measure and Jaccard index use ‘pixel-wise’ comparison, whereas ΔO uses ‘object-wise’ comparison. Although the value of ΔO, by being an absolute difference value, is equally affected by both ‘under’ (false negative) and ‘over’ (false positive) object segmentation, it shows lower accuracy when both phenomena are combined on the same image (since false positives and false negatives will cancel out). For that reason, the first two indicators are more robust when trying to observe small effects between algorithms showing moderate performances (*e.g.* incremental learning). However, we believe ΔO is still a valuable indicator for the screening of very rare crystals, since its ‘binary’ behavior can sense partial segmentation to a higher extent than the other two indicators.

Furthermore, ΔO (normally used on motion segmentation) can be biased by differences in the total number of objects on the ground-truth segmentation (in our case, differences in cell density or crystallization efficiency between images, or between targets). To avoid this, we propose a new indicator that we called ‘normalized Δ object (ΔO*N*)’, calculated as follows:

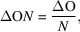

where *N* is the total number of objects from the ground-truth segmentation. This modified indicator shows increased robustness when comparing different experiments.

For all three (*F*, *J* and ΔO*N*) indexes, the range of the values is between 0 and 1 (shown here as percentages). For the first two, higher values indicate a better algorithm performance, whereas for ΔO*N*, the lower the better. All indicators are represented as average values (over all images).

## 3. Results and discussion

GMPR crystals started to grow after 44 h post-infection (p.i.), having a similar rectangular shape as observed for its close structural homolog IMPDH (Nass *et al.*, 2020 ▸), as well as a comparable crystallization efficiency. At day 5 p.i., 95% of the cells in the culture contained a single crystal, protruding from the cellular body, with its longer axis (which can reach up to 140 µm) orthogonal to the light path. The vast majority of the crystals tend to be placed at the bottom of the cells, as observed with confocal optical sectioning (Fig. 2 ▸).

HEX-1 crystals have been observed in the baculovirus-infected cells at day 4 p.i., exhibiting the reported hexagonal cross section and the expected crystallization efficiency (>85%), without protruding from the cell body (Lahey-Rudolph *et al.*, 2021 ▸). As well as the smaller size, HEX-1 crystals show a significant degree of clustering and grow in diverse orientations (Fig. 3 ▸), adding a level of complexity to crystal analysis compared with GMPR, for both manual annotation and automatic segmentation.

The first factor to analyze is the impact of the number of images used to train the Mask R-CNN algorithm, for each respective target (Table 1, rows 1–3 and 10–12; Figs. S1–S3). The results show that just 10 images were sufficient to train the algorithm to recognize each of the targets (Table 1, rows 1 and 10; Figs. S1–S3). We did not observe a very significant improvement of these indicator values with the addition of more images (up to 50), despite the general tendency towards a slight performance improvement. Hence, we chose 50 images as the best compromise, selecting this as a base for the following training strategies. Regarding cell recognition, the model trained with the science-bowl dataset is able to correctly recognize the cell bodies (Fig. 4 ▸) on the basis of previous training approaches (Mela & Liu, 2021 ▸). Our results indicate that learning approaches are useful for recognizing *in cellulo* crystals, using a limited number of epochs and annotated images.

We next investigated the ability of the trained algorithm to recognize crystal targets of a different nature than that of those used for generating the training set. For that purpose, the previously trained algorithms (on H or G targets) were used to recognize the other (G or H, respectively) target crystals. Despite the trained algorithms being able to recognize some of the ‘new’ target features, the quality of the predictions was lower in both cases, as expected (Table 1, rows 1–3 and 10–12; Figs. 4 ▸ and S1–S3).

In light of these results, we tried to follow the incremental learning approach, based on the continuation of the previous training (that was ‘off target’) by adding (10, 30 or 50) annotated images from the target to be tested. In this approach, we set two different learning rates (1 × 10^−3^ and 1 × 10^−4^) to test the influence of this parameter on the outcome.

**Figure 5:**
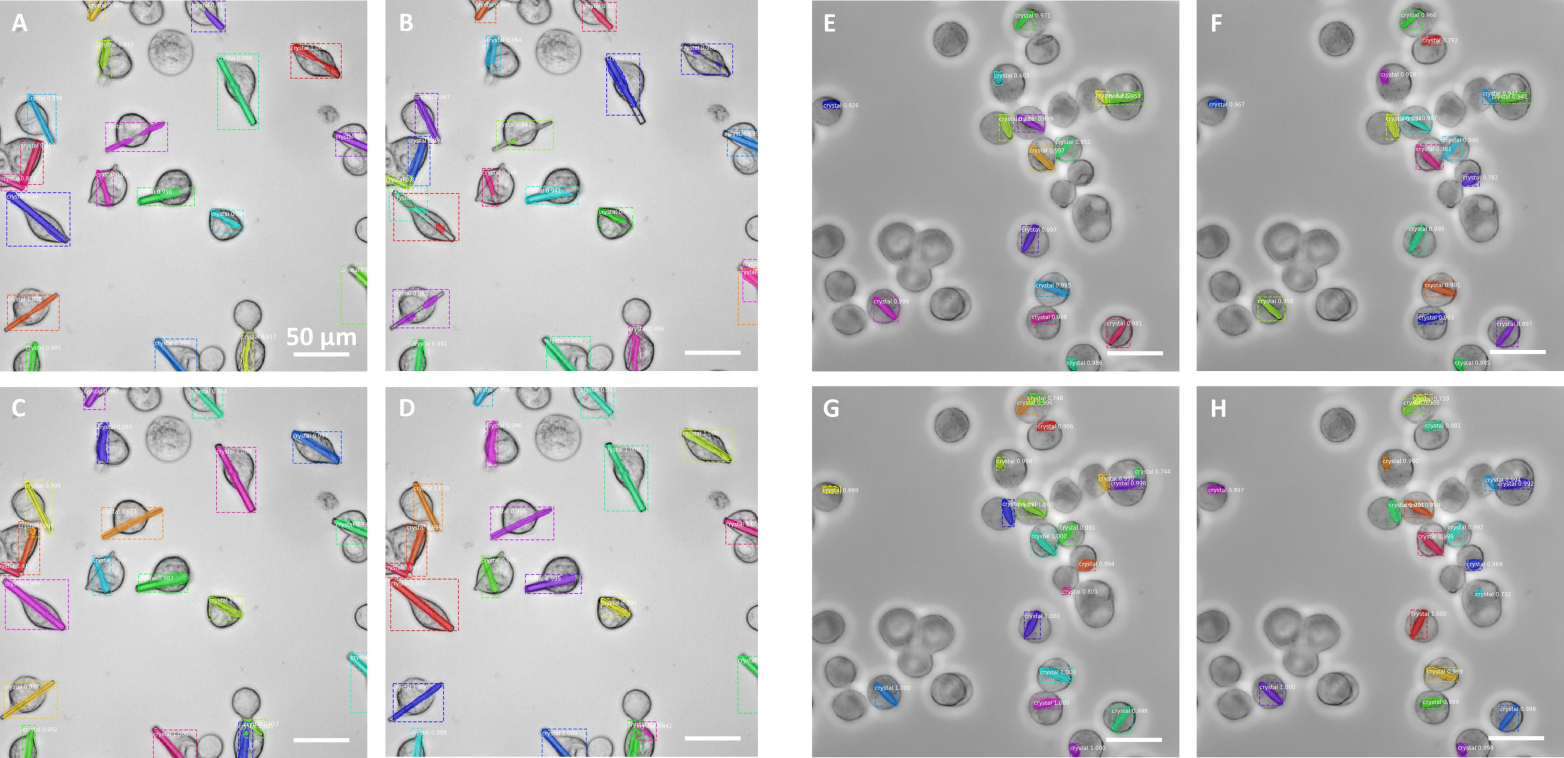
Secondary training of the previous algorithms (G_50_ or H_50_) on ten images of target G (H_50_ > G_10_, top panels A and B, and E and F) or target H (G_50_ > H_10_, bottom panels C and D, and G and H), using 10 epochs and learning rates of 1 × 10^−3^ (panels A and C, and E and G) or 1 × 10^−4^ (panels B and D, and F and H), and evaluation on target G (left, A–D) or H (right, E–H). Whereas G_50_ > H_10_ seems to be performed correctly on both targets (with low influence by the learning rate used), the H_50_ > G_10_ algorithm seems to require higher learning rates for adapting to the secondary target (losing in turn adaptation to its primary target). This shows that the learning rate is a parameter that must be tuned depending on each target’s characteristics. Performance indicator values are shown in Table 1 ▸.

The results (Table 1, rows 4–5 and 13–14; Fig. 5 ▸; Figs. S4–S6) indicate that the addition of these few images (of the target to be tested) to the training process (referred to here as ‘secondary’ training) improved the recognition of this ‘secondary’ target significantly, and for both targets (*e.g.* G_50_ versus G_50_ > H_10_ performance on target H, or H_50_ versus H_50_ > G_10_ performance on target G). A progressive increase of the images used for this ‘secondary’ training did not result in a clear change in performance, suggesting that 10 images are sufficient to generate the observed changes (Table 1, rows 4–9 and 13–18).

However, when looking at the performance of the resulting algorithms on their ‘primary’ targets (*e.g.* G_50_ versus G_50_ > H_10_ performance on target G, or H_50_ versus H_50_ > G_10_ performance on target H), the effect of this ‘secondary’ training substantially differs. Thus, in the case of the algorithm ‘primarily’ trained on target H (H_50_, smaller, more complex crystals), the ‘secondary’ training (H_50_ > G_10_, providing ‘versatility’ towards target G) comes across with a negative impact on the recognition of its ‘primary’ target (*e.g.* *F*-measure of 82.51 versus 63.88, respectively), a consistent behavior observed, to a significant extent, for both performance indicators (Table 1; Figs. S4–S6). These findings suggest that, in some cases, widening the scope of targets to be recognized is at the expense of sacrificing specific performance. On the other hand, this behavior is not observed when G (G_50_, larger, less complex crystals) was the target used for the primary training, suggesting that, in our particular setup, the incremental learning approach is optimal when the primary training is done with simpler cases (*e.g.* larger and less complex targets), but not the other way around. Furthermore, our results showed a differential effect on the incremental learning approach depending on the training strategy used, which needs to be considered when designing the pipeline for new experiments.

In that sense, we also observed that, when this negative impact (of the secondary training in the recognition of the primary target) occurred, it was more evident when higher (1 × 10^−3^) learning rates for the ‘secondary’ training were used (*e.g.* for H_50_ versus H_50_ > G_10_, *F*-measure of 82.51 versus 63.88 for target H), with half of the negative impact observed using lower (1 × 10^−4^) learning rates (*F*-measure of 82.51 versus 73.49) for the same training sets. One possible explanation is that lower (1 × 10^−4^) learning rates (one order of magnitude below those used on the primary training) could have been too low for the secondary training, and therefore showed up in the form of a reduced effect. Interestingly, we found a positive correlation between the intensity of this negative impact (on the primary target segmentation) and the benefits observed on the recognition of the ‘secondary’ target, which were, on the other hand, higher when using higher learning rates (*e.g.* for H_50_ versus H_50_ > G_10_, *F*-measure of 55.77 versus 73.77 for LR = 1 × 10^−3^, and 55.77 versus 62.75 for LR = 1 × 10^−4^, both for target G). These results indicate that, in this particular case, the adaptations to new features came at the expense of prior knowledge (being more evident using higher learning rates for this secondary training), and that a systematic procedure is required to obtain the most from the incremental learning strategy.

**Figure 6:**
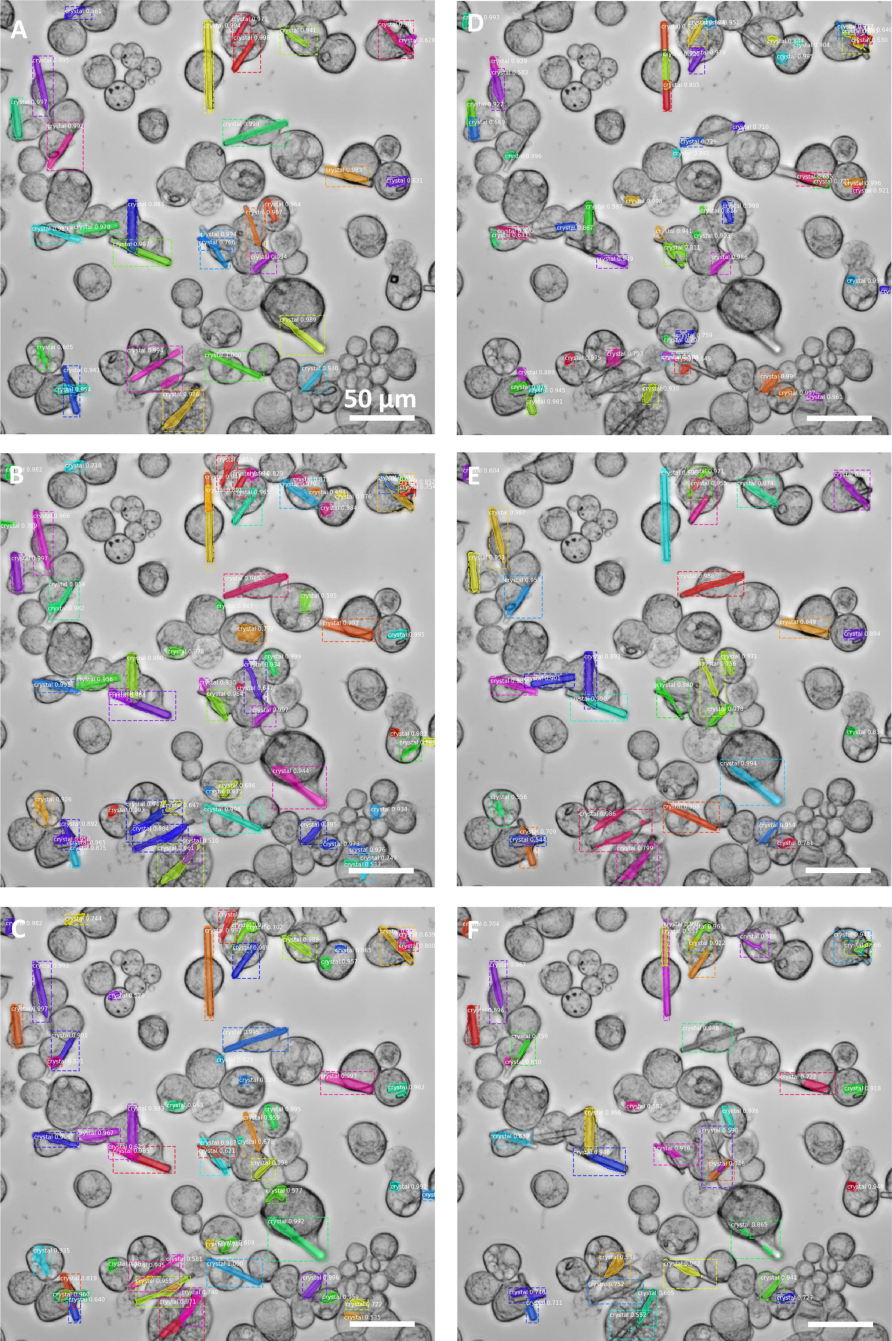
Performance of the different algorithms (Table 1 ▸: rows 3, 8, 9, 12, 17 and 18) on mixed crystal context. Algorithms resulting from both primary (G_50_ or H_50_, panels A and D, respectively) and incremental learning trainings (G_50_ > H_50_, panels B and C; or H_50_ > G_50_, panels E and F) were tested on images containing both targets (even coexisting in the same cell). Secondary trainings were performed using 10 epochs and learning rates of 1 × 10^−3^ (panels B and E) or 1 × 10^−4^ (panels C and F). The H_50_ > G_50_ algorithm seems to require higher learning rates for adapting to the secondary target (losing in turn adaptation to its primary target). Performance indicator values are shown in Table 1 ▸.

To test the algorithm performance in a more complex scenario, images of cells containing both crystal targets were generated by co-infection of the cell culture with the two different viral stocks at equivalent ratios. This approach produced images showing the two different crystal types in a similar proportion and, in some cases, even within the same cell. The predictions made by the various algorithms on these new images (Fig. 6 ▸) were consistent with those made in the context of single crystals (Figs. 4 ▸ and 5 ▸), confirming the increased versatility provided by the secondary training, as opposed to the more specific behavior of the algorithms trained on single targets (Fig. 6 ▸, panels A and D). As the separate annotation of each crystal type cannot be carried out unambiguously in this context, we could not produce performance indicators as done for the other algorithms. Instead, we looked at the prediction box score averages (G_50_ > H_50_ algorithm, LR = 1 × 10^−4^; Fig. 6 ▸, panel C) over a significant (>100) number of crystals from these (two-crystal) images, either present in isolated form (0.914) or coexisting with another crystal type in the same cell (0.897). The results confirmed that the algorithm showed a similar ability to recognize crystals in both cellular contexts. These values were also similar with respect to those obtained (using the same algorithm) from images containing either H (0.951) or G (0.941) crystal types, which showed slightly higher scores that can be explained by these (single-target) images being a much closer match to the training image context.

As previously discussed, the incremental methods, despite providing the best results in this study (Table 1 ▸) increasing the versatility of the algorithm to recognize different targets (Figs. 5 ▸ and 6 ▸), show, in some cases, suboptimal responses depending on the crystal targets used for the different training steps. To address this, in a separate experiment we combined the individual target images used for the incremental learning (50 from each of the two targets) in a single dataset, and used it to train the algorithm in a single step (H_50_ + G_50_), applying the standard parameters used for the primary trainings (40 epochs and LR = 1 × 10^−3^). The algorithm trained following this approach (Table 1 ▸, row 19; Figs. S4–S6) showed a convergence (*F*-measure of 78.09 and 73.85, for H and G targets, respectively) towards the best performing algorithms, obtained with some of the (generally faster) incremental learning methods, thus constituting an alternative to those cases where the latter fails to produce the best results.

Note that, while having a negative impact in some cases, the secondary training did not seem to benefit, for either of the two crystals, the performance of the algorithm on the recognition of its primary target. This, on the one hand, demonstrates that no additional useful information is obtained from the training on a different crystal type and, on the other hand, indicates that the algorithm is very efficient in extracting all the necessary information from very few images.

Investigating the cause of this variability on the secondary training response by the algorithm is outside the reach of the present article, but a possible explanation could relate to the fact that the targets used in the primary and in the secondary training differed in size (and/or complexity). In such a scenario, the primary training (with 50 images) on the smaller (and complex) target (*e.g.* H_50_ algorithm) led to ‘over segmentation’ (multiple segments for a single crystal) of the ‘bigger’ (G target) objects, with each segment corresponding to the size of the smaller target (panels D in Figs. 4 ▸ and 6 ▸). In extreme cases, it resulted in a higher number of predicted objects than ground-truth objects, as observed for some of the images (Fig. 4 ▸, panel D). This incomplete/partial segmentation is reflected in a negative impact on all three performance indicators. On the other hand, a primary training (with 50 images) on the larger (less complex) target (*e.g.* G_50_ algorithm) did not show this ‘mosaic’ segmentation on the smaller (target H) objects, despite resulting in equally poor predictions with a higher percentage of missed objects (Fig. 4 ▸, panel G) and similarly low performance indicator values. These observations may be an indication that the underlying reasons (for the lower ‘off-target’ performance) could be essentially different in both cases. And this could in turn explain why, when we apply the incremental learning methods, the algorithm responds differently to this secondary training, in each of the targets, and why the learning rate may play a differential role. According to our results, the use of a single training step, with a combination of images of both targets, represents an alternative to the incremental learning strategy.

Altogether, the results confirmed the effortless adaptability of the algorithm, and the potential to improve it with every new target studied. However, this incremental learning approach, in some cases, may interfere with the previous knowledge of the algorithm, causing it to ‘unlearn’, improving the versatility while reducing specificity. The current article sheds some light on this behavior, and indicates that the approach requires tuning depending on the characteristics of the objects used for training the algorithm.

## 4. Conclusions

We showed the potential of our method for the segmentation of *in cellulo* crystals. Further, we propose a model based on Mask R-CNN, which accurately detects different types of intracellular crystals, with well differentiated shapes. The model can be further tuned and effortlessly adapted to new crystal shapes. In addition, optical sectioning allows us to segment the cells and crystals in different layers, widening the scope to three-dimensional segmentation of the objects.

The current setup, including the fully automated acquisition, is intended to be used as a general screening pipeline to rapidly score cell cultures for successful intracellular crystal growth. It is particularly useful for cells producing unknown protein targets with low or very low crystallization efficiency, preventing hours of manual cell scoring. The algorithm can also assist in the selection of optimization protocols during *in cellulo* crystallization, being able to monitor the impact of the different approaches on the occurrence or the size or shape of the crystals. Potentially, it can be used to perform real time tracking of cells containing crystals (or isolated crystals) during X-ray diffraction experiments at synchrotrons or free-electron lasers, either placed on fixed targets or flowing through high-viscosity jets. This could result in significant advantages like avoiding the need to irradiate the sample for target localization, or synchronizing beam exposures to selected targets or crystal shapes.

## Supplementary Material

Supporting figures. DOI: 10.1107/S1600576724000682/jo5091sup1.pdf

Repository containing all files required to complement the manuscript: https://doi.org/10.5281/zenodo.10475961

## Figures and Tables

**Table 1:** Validation of the model The quantitative results of the testing are reported on the basis of the three different performance indicators, for both H and G targets. The original values (0 to 1 range) are represented here as percentages (0 to 100 range). Higher values of *F*-measure and Jaccard index, or lower values of ΔO*N*, indicate a better performance. In the second column from the left, the different (primary > secondary) training strategies (with target and number of images used) are represented. A graphical representation of Table 1 ▸ can be seen (in a digest format) in Figs. S1–S6. Row numbers are indicated (left column). LR = learning rate.

No.	Training set (primary > secondary)	Average *F*-measure (%) target H/target G	Average Jaccard index (%) target H/target G	Average ΔO*N* (%) target H/target G
1	G_10_ > –	47.14/75.67	31.41/60.94	67.49/17.79
2	G_30_ > –	58.95/77.11	42.23/62.82	53.93/15.97
3	G_50_ > –	54.95/77.70	38.38/63.60	61.28/15.76
4	G_50_ > H_10_ (LR = 1 × 10^−3^)	82.35/75.34	70.13/60.52	16.49/14.53
5	G_50_ > H_10_ (LR = 1 × 10^−4^)	78.79/77.23	65.18/62.97	17.86/11.52
6	G_50_ > H_30_ (LR = 1 × 10^−3^)	81.76/76.20	69.27/61.63	14.60/17.02
7	G_50_ > H_30_ (LR = 1 × 10^−4^)	79.92/77.34	66.69/63.12	17.34/10.66
8	G_50_ > H_50_ (LR = 1 × 10^−3^)	83.34/75.64	71.55/60.90	13.77/22.79
9	G_50_ > H_50_ (LR = 1 × 10^−4^)	80.93/76.89	68.11/62.52	17.19/13.02
10	H_10_ > –	82.19/54.40	69.92/37.65	16.25/25.01
11	H_30_ > –	81.61/55.07	69.10/38.36	13.69/43.23
12	H_50_ > –	82.51/57.77	70.38/40.97	11.35/50.32
13	H_50_ > G_10_ (LR = 1 × 10^−3^)	63.88/73.77	47.41/58.53	49.87/19.34
14	H_50_ > G_10_ (LR = 1 × 10^−4^)	73.49/62.75	58.36/45.85	30.69/11.33
15	H_50_ > G_30_ (LR = 1 × 10^−3^)	67.49/73.01	51.26/57.61	38.16/13.90
16	H_50_ > G_30_ (LR = 1 × 10^−4^)	73.65/60.29	58.57/43.30	30.75/13.25
17	H_50_ > G_50_ (LR = 1 × 10^−3^)	68.36/74.26	52.33/59.16	44.67/19.40
18	H_50_ > G_50_ (LR = 1 × 10^−4^)	73.47/59.93	58.38/42.94	29.89/12.85
19	H_50_ + G_50_ (LR = 1 × 10^−3^)	78.09/73.85	64.26/58.64	21.04/16.13
