# Supplementary material for: Convolutional neural network approach for the automated identification of in cellulo crystals
Source: J Appl Crystallogr. 2024 Feb 23;57(Pt 2):266–75. doi: 10.1107/S1600576724000682 (PMC11001417; doi:10.1107/S1600576724000682)
Supplement: Supplementary file 1 [file j-57-00266-sup1.pdf]

JOURNAL OF APPLIED CRYSTALLOGRAPHY

Volume 57 (2024)

Supporting information for article:

Convolutional neural network approach for the automated identification of *in cellulo* crystals

Amirhossein Kardoost, Robert Schönherr, Carsten Deiter, Lars Redecke, Kristina Lorenzen, Joachim Schulz and Iñaki de Diego

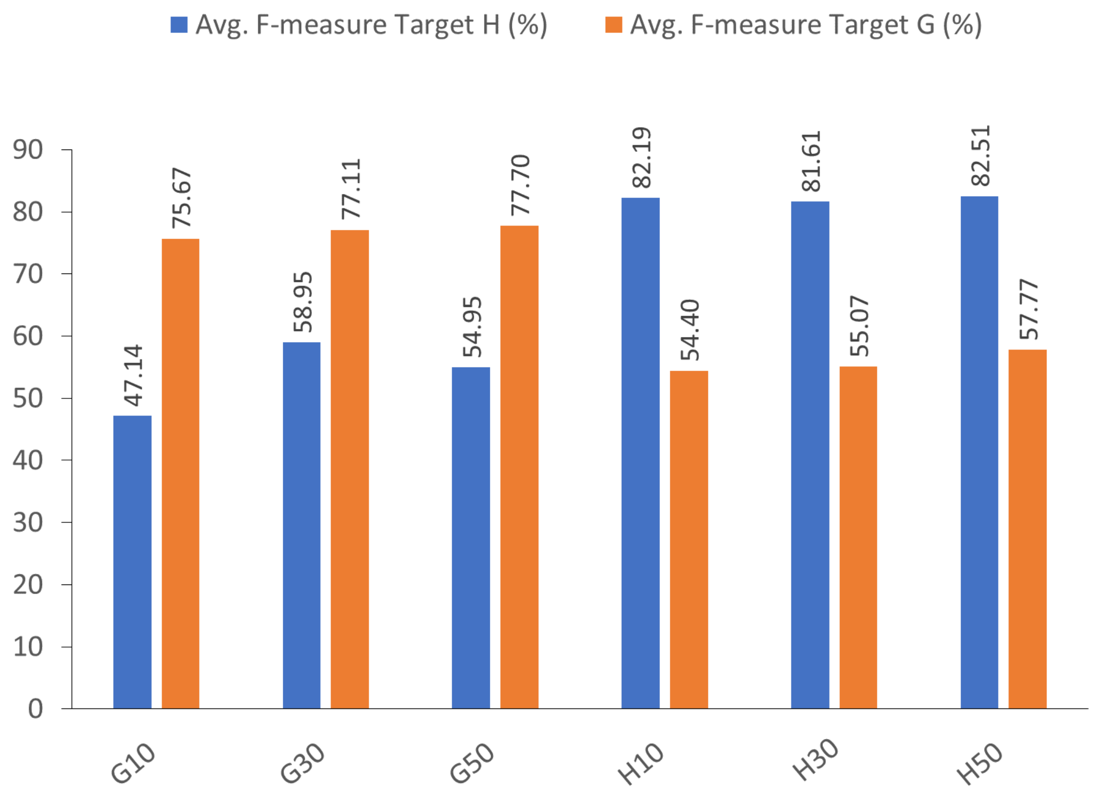

Figure S1 Average F-measure Index showing the effect of the primary training on the recognition of each of the targets. The different training strategies are indicated on the X axis (showing target followed by number of images of the training set). On the Y-axis, the values of F-measure indicated in Table 1, are represented. Different color bars represent each of the targets on which the algorithm was tested. A higher value of F-measure corresponds to better performance. Values are shown as percentages.

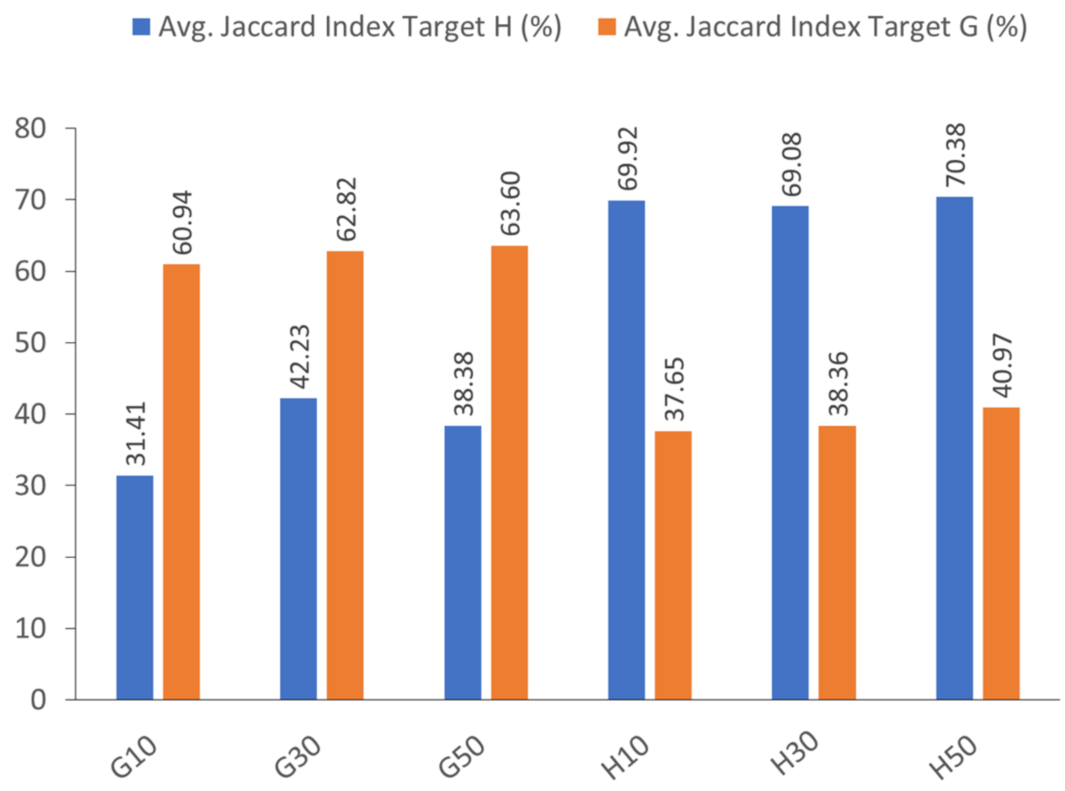

Figure S2 Average Jaccard Index showing the effect of the primary training on the recognition of each of the targets. The different training strategies are indicated on the X axis (showing target followed by number of images of the training set). On the Y-axis, the values of the Jaccard Index indicated in Table 1, are represented. Jaccard Index allows to estimate shape-matching accuracy, so higher values correspond to better performance. Values are shown as percentages.

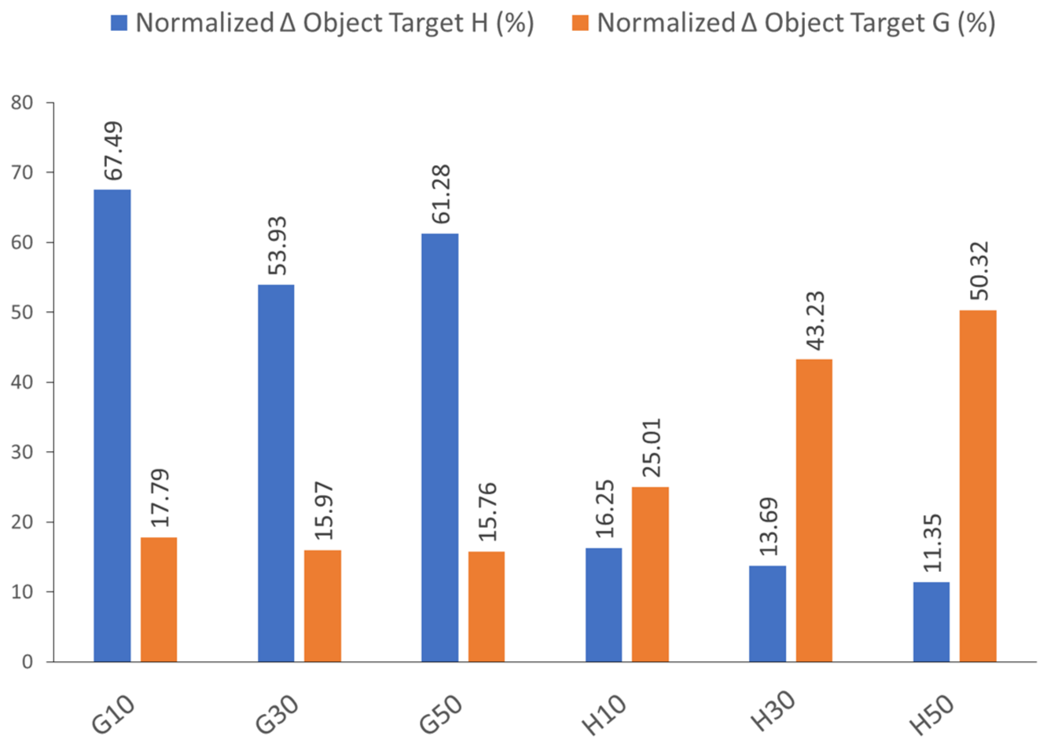

Figure S3 Average Normalized Δ Object showing the effect of the primary training on the recognition of each of the targets. The different training strategies are indicated on the X axis (showing target followed by number of images of the training set). On the Y-axis, the values of the Normalized Δ Object indicated in Table 1, are represented. Normalized Δ Object is an indicator of the absolute difference between ground truth and predicted objects, divided by the number of ground truth objects, so lower values correspond to better performance. Values are shown as percentages.

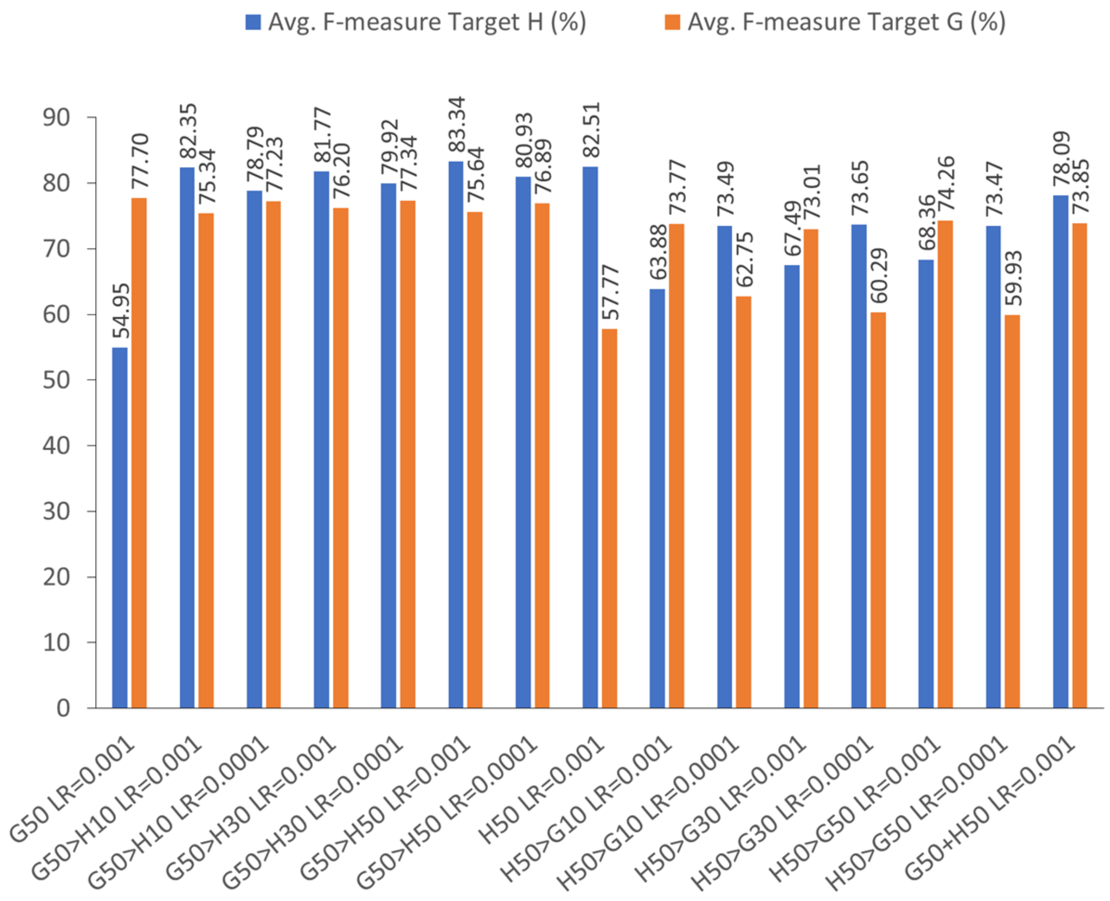

Figure S4 Average F-measure Index showing the effect of the secondary training on the recognition of each of the targets. The different training strategies are indicated on the X axis (showing primary>secondary targets followed by number of images on each training subset). On the Y-axis, the values of F-measure indicated in Table 1, are represented. Different color bars represent each of the targets on which the algorithm was tested. A higher value of F-measure corresponds to better performance. Values are shown as percentages.

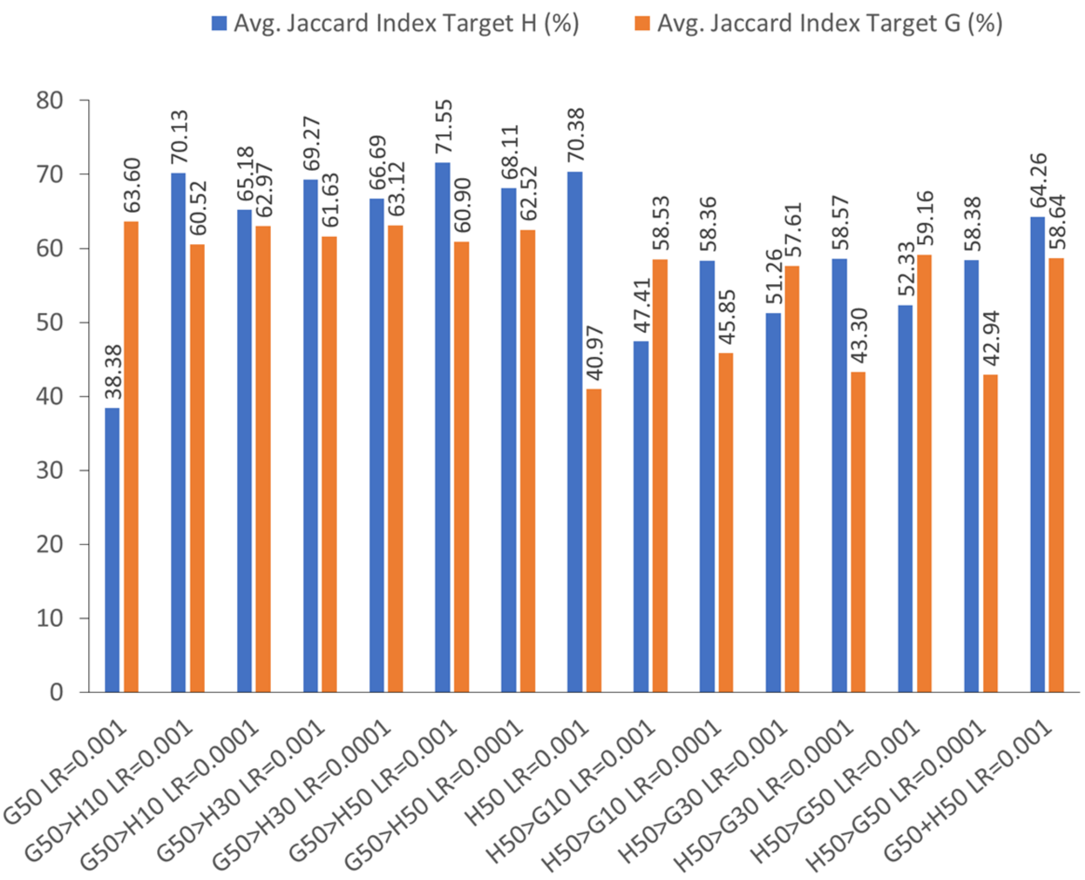

Figure S5 Average Jaccard Index showing the effect of the secondary training on the recognition of each of the targets. The different training strategies are indicated on the X axis (showing primary>secondary targets followed by number of images on each training subset). On the Y-axis, the values of the Jaccard Index indicated in Table 1, are represented. Jaccard Index allows to estimate shape-matching accuracy, so higher values correspond to better performance. Values are shown as percentages.

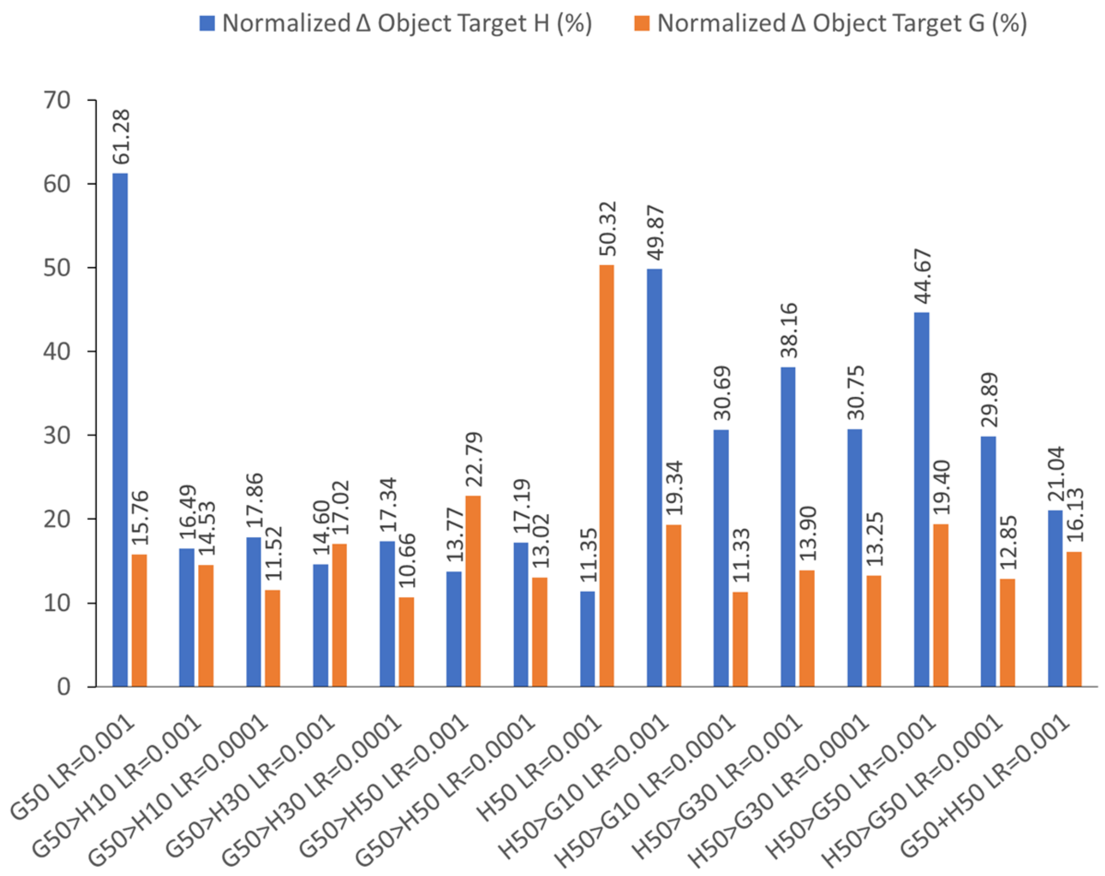

Figure S6 Average Normalized Δ Object showing the effect of the secondary training on the recognition of each of the targets. The different training strategies are indicated on the X axis (showing primary>secondary targets followed by number of images on each training subset). On the Y-axis, the values of the Normalized Δ Object indicated in Table 1, are represented. Normalized Δ Object is an indicator of the absolute difference between ground truth and predicted objects, divided by the number of ground truth objects, so lower values correspond to better performance. Values are shown as percentages.
